# The Advent of Old Age

**Published:** 1873-09

**Authors:** 


### THE ADVENT OF OLD AGE.

THERE is a certain period in life when the human body, as well as mind, reaches its maximum point of development, and from that point to the grave is the season of decay. This idea is far more forcibly expressed by Shakspeare, who makes Jacques, in "*As You Like It*," say:

> "And so, from hour to hour we ripe and ripe,
> And then from hour to hour we rot and rot,
> And thereby hangs a tale."

At what age are we to look for this change? The Psalmist says the days of man are three score and ten, and some of our readers, perhaps, will remember a pretty little poem of Willis', written on his thirty-fifth birthday, begining: "I'm half way home." But poets are not very reliable authority in matters of science. A distinguished French physiologist, writing upon this subject, says: "I propose the following natural divisions and natural durations for the whole life of man:

The first ten years of life are . Infancy.
" second " " . . Boyhood.
" third " " . . First Youth.
" fourth " " . . Second Youth.
From forty to fifty-five, . . First Manhood.
From fifty-five to seventy, . . Second Manhood.

And this period of manhood is the age of strength, the manly period of human life.

From seventy to eighty-five, . . First Old Age.
From eighty-five to one hundred, . Second Old Age.

These deductions are made from a careful study of the question, with all the aids derivable from a thorough knowledge of the sciences of anatomy and physiology. It is not claimed but what these divisions will vary in different individuals, and overlap each other in the same one; but that they are as correct as such a general truth can be stated we verily believe. These limits are not so arbitrary as they may seem at first sight. At ten years of age the second toothing is completed and infancy ought to end; at twenty the bones no longer increase in length and boyhood naturally ends; at forty the body ceases to increase in size and youth ends, and so on. After forty whatever increase there may be of the body is in fat; and, instead of increasing its strength and activity, this latter growth weakens the body and retards its motions. When the growth ceases absolutely the body rests, rallies, and becomes invigorated. This period of internal invigoration is the period of the first manhood, and lasts fifteen years, and maintains itself fifteen years longer, when the period of old age begins; and this period begins when we have no longer any reserve of strength to draw upon, and when the natural strength is barely sufficient for the daily work, and when anything unusual fatigues, and extraordinary efforts impair the general health. When this condition of things arrives old age has fairly begun, *and this period is at seventy years of age.*

Buffon, the distinguished naturalist, says "that the man who does not die by accident or disease lives to ninety or a hundred years." That he is eminent authority no one will dispute. It is very true, however, that comparatively few men live to be ninety or a hundred years old, but that affords no argument against the truth of our proposition. Most men die of disease—only a very few of old age. The death record of any place will show, however, quite a respectable number of people who live to between eighty and one hundred years. The rector of Trinity Church, Brooklyn, attended the funeral of an elderly lady, a member of his congregation, one day this winter, and he said, in course of his remarks, that that was the third funeral he had attended that week, and that the united ages of the three defunct would be over 250 years! If any considerable number of people live to this age, it is an unanswerable argument in support of the opinion that all, or at any rate most people may. But is length of days desirable? We may, in a subsequent number, answer this question.

